# Low‐level laser therapy using laser diode 940 nm in the mandibular impacted third molar surgery: double-blind randomized clinical trial

**DOI:** 10.1186/s12903-021-01434-1

**Published:** 2021-02-18

**Authors:** Ehsan Momeni, Hoda Barati, Melika Rajaei Arbabi, Behrouz Jalali, Mahdieh-Sadat Moosavi

**Affiliations:** 1 Department of Oral Medicine, School of Dentistry, Arak University of Medical Sciences, Arak, Iran; 2 School of Dentistry, Arak University of Medical Sciences, Arak, Iran; 3 Department of Oral Surgery, School of Dentistry, Arak University of Medical Sciences, Arak, Iran; 4 Laser Research Center of Dentistry, Dentistry Research Institute, Tehran University of Medical Sciences, Tehran, Iran; 5 Department of Oral Medicine, School of Dentistry, Tehran University of Medical Sciences, Tehran, Iran

**Keywords:** Low‐level laser, Pain, Surgery, Third molar

## Abstract

**Background:**

The effect of low-level laser therapy (LLLT) on pain mitigation following the third molar surgery is still controversial. The absence of a standard method for using laser among the studies is related to the types of sample selection, sample size, control, and LLLT parameters, which make pain mitigation following surgery more controversial. Therefore, this study aimed to determine the effect of LLLT on reducing pain, swelling, and trismus following the mandibular impacted third molar surgery.

**Methods:**

This study was performed on 25 healthy subjects. After the surgery, amoxicillin 500 mg was prescribed every 8 h for a 7-day period besides oral Ibuprofen (Gelofen) 400 mg every 12 h for a 3-day period. The intraoral Laser diode 940 nm was applied immediately after suture on the tested side, while on the placebo side, a fiber tip was used with no laser radiation following surgery. Eventually, the pain score was evaluated by VAS index from the 1st to the 7th-day post-surgery and then analyzed by SPSS 24.

**Results:**

The results indicate that the mean swelling and trismus before, during, 2 days after, and 7 days after the intervention did not differ significantly between the two studied groups. However, the results show that on the sixth and seventh days, the pain was significantly lower in the intervention group compared to the control group.

**Conclusions:**

The results suggest that although the pain, swelling, and trismus following surgery were lower on the radiated side, only pain was found to be significant on the radiated side (*p* < 0.05).

The registration number of the clinical trial in a Primary Registry in the WHO Registry Network is IRCT20141209020258N110 and the date of retrospective registration is 04/05/2019. The related URL is https://www.irct.ir/trial/36321.

## Background

Surgical extraction of mandibular impacted third molars is a typical dental surgery [1–5]. This surgery may result in postoperative pain because of tissue damage and inflammation [6, 7]. Following surgery, despite using proper principles of patient’s preparation, novel techniques in surgery, and precise controlling of soft and hard tissues that can reduce postoperative complications; certain inevitable complications would still occur. The factors affecting the development of these complications are complex and mostly associated with swelling, which is caused by surgical trauma. Notably, pain, swelling, and constrained mouth opening are recognized as the most common postoperative problems [8]. Pain increases within the first 5 h and then diminishes until the end of the first-week post-operation [2, 4, 7]. Pain management following surgery was reported via the consumption of some drugs such as Nonsteroidal anti-inflammatory drugs (NSAIDs) and corticosteroids [2, 9]. Nevertheless, their consumption is often associated with several side effects, including stomach ulcers, gastrointestinal bleeding and perforation, impaired renal function, allergic reactions, and the inhibited function of platelets [9]. To minimize these side effects and to mitigate pain following surgery, alternative methods are used such as surgical closure methods with or without drain connection, cryotherapy, and laser [10, 11].

Low-level laser therapy (LLLT) was discovered by chance in an attempt to kill cancer cells using a laser [12]. It was found that it does not kill the tumor cells, rather it promotes the process of wound healing [12]. Since then laser is used in dentistry for different purposes including ulcer healing [13–15], aphthous stomatitis [13], mucositis, neural regeneration, post herpetic neuralgia, synovitis, arthritis, problems of the temporomandibular joint, acute swelling, periapical granuloma, gingival depigmentation [15], chronic orofacial pain [14], and bone regeneration [13]. The analgesic effect of LLLT by stimulating the synthesis of endogenous endorphins (beta-endorphin), reducing inflammatory cytokines and enzymes, altering the pain threshold, inducing changes in morphological neurons, reducing the mitochondrial membrane potential, and blocking rapid axon flow, consequently causes neural conduction blockade [2, 16]. The anti-inflammatory effect occurs due to the increased phagocytic activity [16], the number and the diameter of lymphatic vessels [16], the diminished permeability of blood vessels and microcapillary blood circulation restoration [16], normalization of blood vessel permeability [16], and the diminished edema [17]. The effect of LLLT on third molar postoperative pain is still controversial. A study has reported no beneficial effect with an 830 nm laser on swelling, trismus, and pain after the third molar surgery [18]. Some studies have shown a clinical significance in postoperative pain and swelling when using LLLT [9, 19]. El-sound has found that the pain level in the laser group (870 nm wavelength laser for non-impacted third molar extraction) was lower than in the placebo group throughout the 7-day follow-up period [9]. In another study by Batinjan, it was shown that after the impacted third molar surgery, patients who received photodynamic therapy (laser with a wavelength of 660 nm) experience lowered temperatures and less wound swelling in this regard [19]. Moreover, using a diode laser with a wavelength of 660 nm with 5 J/cm^2^ for 8 s for four consecutive daily sessions suggested a significant clinical effect on pain and swelling [16]. In another study, the use of a 904 nm laser prevented the increased interleukin (IL)-1β, IL-6, IL-10, and cyclooxygenase-2 (COX-2). Moreover, the patients reported less pain at the laser-treated site compared to the pain reported at the control site [20].

The absence of a standard method for using laser among studies is related to sample selection, sample size, control, and parameters of LLLT, which consequently cause controversy in postoperative pain mitigation. One of the used wavelengths in LLLT is 940 nm [21]. However, there are still conflicting evidences on its effects on the third molar surgery [22]. Since the possibility of clinical use is important, in the present study, laser treatment was used for one session on the same day of surgery. Additionally, to benefit from the specific local anti-inflammatory effects, unlike the previous studies [22], in the current study, laser irradiation was intraorally performed at the surgical site. So, this study aimed to investigate the impact of intraoral LLLT using laser diode 940 nm on pain, swelling, and trismus following the surgical extraction of the mandibular impacted third molar.

## Methods

### Trial design

This study was performed as a double-blind randomized clinical trial. The registration code of the clinical trial in a Primary Registry in the WHO Registry Network is IRCT20141209020258N110. The present study was performed after obtaining approval from the University Ethics Committee (Code: IR.ARAK.REC.1397.158). Accordingly, all the procedures were accomplished in terms of the associated instructions.Written informed consent was taken from all the patients.

### Sample size

The sample size was calculated based on the previous studies (α = 0.05, β = 0.2, Mean in group 1(µ_1_) = 2.77, Standard deviation in group 1(σ_1_) = 1.45; Mean in group 2(µ_2_) = 4.11, Standard deviation in group 2(σ_2_) = 1.41, r = 1) [23].

$$n\geq\frac{\left(Z_{1-\frac\alpha2}+Z_{1-\beta}\right)^2\left(\sigma_1^2+\frac{\sigma_2^2}r\right)}{{(\mu_1-\mu_2)}^2}$$

### Participants

Patient recruitment was done over 3 months. Each patient had two follow-ups once on the second and once on the seventh day after surgery. Twenty-five healthy subjects aged between 18 and 40 years old with asymptomatic mandibular impacted third molars were included in this study. In terms of impaction, the teeth should be in Class A according to Pell and Gregory’s classification. The exclusion criteria were as follows: a systemic disease, chronic pain, neurological or psychiatric disorders, photosensitivity, and sensitivity to local anesthesia. Furthermore, the exclusion criteria were acute pericoronitis and periodontal disease, pregnancy, breastfeeding, smoking, and the consumption of analgesic or anti-inflammatory drugs 2 weeks prior to the study. Before the surgery, the gender and age of the patients were recorded.

### Randomization

The side on which the surgery was done was randomly specified in the participants of the case and control groups using computer software (Random Allocation Software with blocked randomizations setting) [24].

### Blinding

This study was a double-blind study, in which neither the patient nor the surgeon and the researcher who measured the outcomes after LLLT were aware of the side that had received treatment. Only the operator who had applied the laser was aware of this information. The noise of the device was cut off during the exposures, so the patients were blinded to the side allocations.

The allocated groups were recorded on sealed and opaque envelopes. These cards were prepared by an independent person who was not involved in the study protocol. To explain, once the patient underwent tooth extraction and agreed to participate in this trial, the allocation assignment was revealed by opening the envelope by this independent person.

### Interventions

An oral surgeon performed both operations of mandibular impacted wisdom teeth with a 3-week interval on both sides for the patients.

The right and left mandibular third molar surgeries were done using the standard technique. The patients received anesthesia of alveolar, lingual, and buccal nerve blockades using two 1.8 ml carpools containing lidocaine 2% with epinephrine 1:8000 (New Colombia, Guarne, Stetic USA). An incision was made by the use of Bromed blade No. 15 (Ontario, USA) whereby the complete mucoperiosteal flap was prepared which was then repositioned using the periosteal elevator Malt 9 (Illinois, Nordent, USA). The buccal and distal bone was removed using carbide round bur 8 Dentsply (Ballaigues, Switzerland) connected to a surgical low-speed hand piece NSK (Tokyo, Japan). Thereafter, the teeth were divided into several parts using a fissure bur Dentsply 703 (Ballaelues, Switzerland) connected to a low-speed handpiece using NaCl saline solution 0.95. Finally, the wound suture was done using nonabsorbable silk fiber 0.3 that was attached to the reversed cutting round needle 3/8 (SMI, Steinberg, Belgium).

**Fig. 1:**
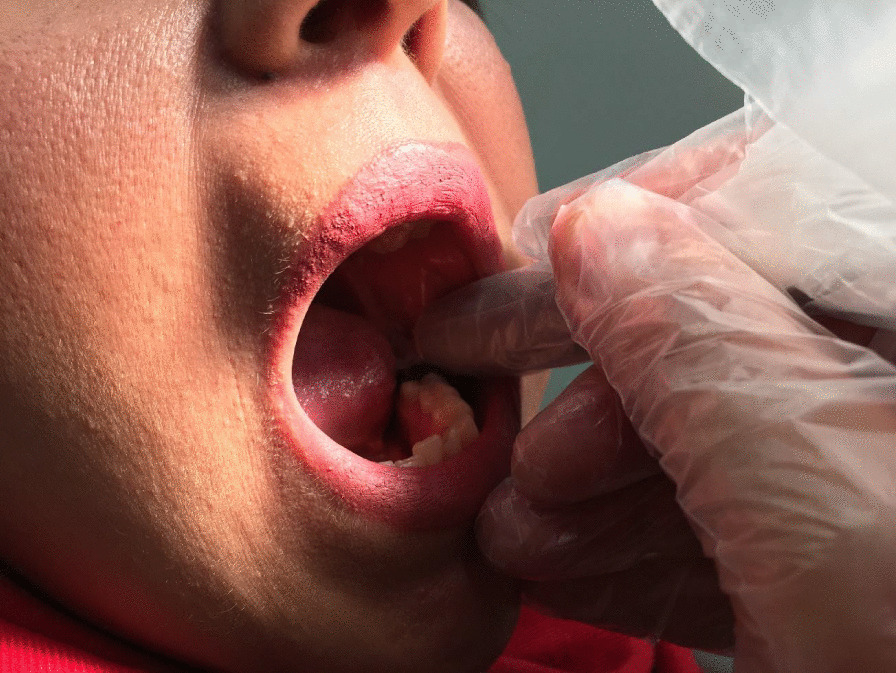
The radiation applied in this research was 940 nm wavelengths. The fiber tip was placed close to the soft tissue and applied for 30 s at three occlusal, buccal, and lingual points with a total time of 90 s and total energy density of 30 J/3 cm^2^ 10

Following performing the surgery, amoxicillin 500 mg was prescribed every 8 h for a 7-day period along with oral ibuprofen 400 mg (Gelofen) every 12 h (every 8 h, if needed) for a 3-day period. The patients were asked to record the number of ibuprofen consumed by them. A trained dental assistant applied the intraoral laser diode 940 nm (BIOLASE) immediately after the suture on the experimented side. On the placebo side, after surgery, only the fiber tip was used without radiation. The laser parameters are shown in Table 1. The fiber tip was placed close to the soft tissue, which was then applied for 30 s at three occlusal, buccal, and lingual points with a total time of 90 s and a total energy density of 30 J/3 cm^2^. Thereafter, the laser was used as a noncontact continuous wave (Fig. 1).

**Table 1:** The laser parameters

Parameter	Laser group
Wavelength (nm)	940
Application/point time (s)	30
No. of application points	3
Density of energy/point (J/cm^2^)	10
Power (W)	0.5
Total intensity in the treated area (J/cm^2^)	30
Application technique	Non-contact

### Outcomes

In this study, the primary outcomes were swelling, trismus, and pain; and the secondary ones were the number of analgesics consumed after the surgery.

**Fig. 2:**
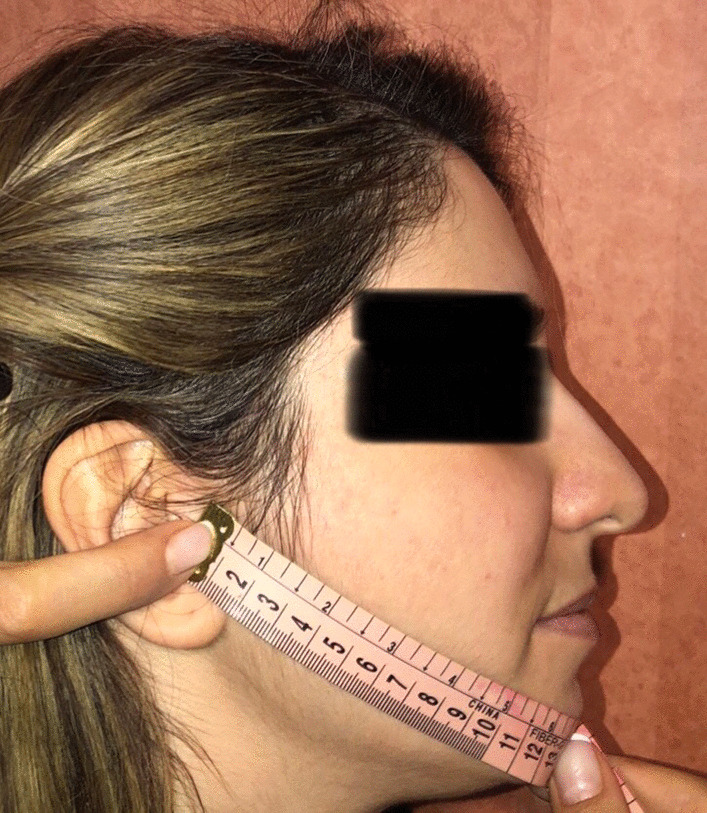
Measuring postoperative swelling

The pain intensity was recorded using a visual analog scale based on its scores (zero as pain-free, and 10 as the worst pain). The patients were given a data recording sheet. The pain scores were recorded every day in a data sheet by the patients from the first day until the seventh-day post-surgery. The extent of mouth opening and swelling was evaluated by an examiner before, immediately after that, and on the second and the seventh days post-surgery. The extent of mouth opening was evaluated by measuring the maximum distance between the central teeth of the mandible and maxilla using a caliper [25]. The extent of swelling was also evaluated by the method of Markovic, and Todorovic [26], which measures the distance between the chin and tragus of the ear (Fig. 2).

### Statistical method

The obtained data were entered into SPSS 24. Descriptive statistics were reported based on central indicators, distribution, frequency, and percentage. Chi-square and independent t-test were used to compare sex and age distributions in the two groups respectively. An ANOVA with repeated measures was also used to perform intergroup and intragroup comparisons of the outcomes of this study

## Results

In this study, 25 patients were included to examine the effect of intraoral LLLT with the wavelength of 940 nm on pain, swelling, and trismus following surgical extraction of mandibular impacted wisdom teeth. The mean age of the patients was 24.08 ± 3.26 years old. Out of them, 15 (60%) subjects were women and 10 (40%) subjects were men. The number of analgesics consumed until the third day had no significant difference between the two groups, which is shown in Table 2.

**Table 2:** The number of analgesics consumed until the third day in the two groups

	Laser	Placebo
Number of analgesics (mean ± SD)	8 ± 3.07	8.66 ± 3.87
*p* value	0.513	

The results indicate that the mean swelling before, during the study, 2 days after, and 7 days after the surgery did not differ significantly between the two groups (*p* > 0.05), as shown in Tables 3 and 4.

**Table 3:** Comparison of swelling in the two groups

Swelling	Laser		Placebo		*p* value
	Mean	SD	Mean	SD	
Before surgery	150.4	9.00	151.2	7.53	0.474
Immediately after surgery	152.4	8.79	152.2	7.51	0.889
2nd day after surgery	156.8	9.11	159.72	7.84	0.174
7th day after surgery	150	7.5	153.2	6.43	0.112

**Table 4:** Confidence intervals in swelling

	Mean ± SD	95% CI	
		Upper limit	Lower limit
Laser	152.4 ± 1.55	155.517	149.283
Placebo	154.08 ± 1.55	157.197	150.963

The results indicate that the mean trismus before, during the study, 2 days after, and 7 days after the surgery did not differ significantly between the two groups (*p* > 0.05). Accordingly, these results are shown in Tables 5 and 6.

**Table 5:** Comparison of trismus in the two groups

Trismus	Laser		Placebo		*p* value
	Mean	SD	Mean	SD	
Before surgery	46.52	4.28	46.96	5.02	0.594
Immediately after surgery	21.92	18.03	25.56	15.96	0.454
2nd day after surgery	29.44	12.59	24.08	9.6	0.097
7th day after surgery	40.2	11.39	34.48	9.8	0.063

**Table 6:** Confidence intervals in trismus

	Mean ± SD	95% CI	
		Upper limit	Lower limit
Laser	34.250 ± 1.77	38.096	30.944
Placebo	32.770 ± 1.77	36.346	29.194

The results show that the mean pain did not differ significantly up to the fifth day. However, on the sixth and seventh days, it was significantly lower in the intervention group (*p* < 0.05) (Tables 7, 8). Notably, no side effects were found in the laser and placebo groups.

**Table 7:** Comparison of pain scores in the two groups

Pain (days after surgery)	Laser		Placebo		*p* value
	Mean	SD	Mean	SD	
1th	5.652	3.12	7.047	2.26	0.119
2nd	4.956	3.12	6.333	2.67	0.129
3th	3.478	2.99	4.857	2.79	0.198
4th	2.782	2.48	4.09	3.08	0.158
5th	1.913	2.31	3.47	2.8	0.083
6th	1.087	1.10	2.76	2.82	0.011*
7th	0.434	1.5	1.714	2.26	0.023*

*p value < 0.05

**Table 8:** Confidence intervals in pain

	Mean ± SD	95% CI	
		Upper limit	Lower limit
Laser	2.901 ± 0.453	3.815	1.986
Placebo	0.427 ± 1.77	5.283	3.370

## Discussion

The present study showed the effect of intraoral low-level laser (500 mw) with a wavelength of 940 nm and an energy density of 10 J/cm^2^ on mitigating pain, swelling, and trismus resulting from the surgical operation of the mandibular wisdom tooth in one session. The results of this study indicate that pain, swelling, and trismus were postoperatively less on the radiated side, but the pain reduction was significant on the sixth and seventh days. It is noteworthy that trauma during surgery to the oral cavity can always cause tissue injury that can be identified by hyperemia, vasodilation, the increased capillary permeability, and infiltration of granulocytes and monocytes. These processes consequently lead to some clinical symptoms such as pain, swelling, and the decreased rate of mouth opening [27, 28].Chronic pain is defined as pain that persists for more than 12 weeks [29]. Postoperative pain is a complicated response to the tissue injury caused by the procedure that hypersensitivity stimulates the central nervous system [30]. In the current study, inflammatory pain was evaluated 7 days after the surgery. It was found that photo radiation can modulate inflammatory pain by reducing levels of biochemical markers (PGE 2, mRNA Cox 2, IL-1beta, and TNFalpha), oxidative stress, and neutrophil cell influx [30]. Some studies have concluded that low-level laser is effective in reducing pain and swelling [31]. However, some others have not mentioned the effectiveness of low-level laser for mitigating pain and swelling [7, 10, 18, 32]. Furthermore, several studies have evaluated the effect of low-level laser on pain mitigation postoperatively in several sessions, but they failed to observe any significant difference in pain mitigation on the placebo and laser sides [2, 4, 33]. The results of the present study are in line with the findings of research in which laser diode 810 nm with an energy density of 32 J/cm^2^ was used to reduce pain [25]. Although in the current study, a lower energy density (10 J/cm^2^) was used, the same reduced pain outcome was obtained on the laser side which was statistically significant. In this study, although low-level laser radiation was done in one session, it had a positive effect on reducing trismus, which could be attributed to the minimum thermal effect occurring during laser radiation that consequently, diminishes trismus. Since the internal pterygoid muscle is exposed to the radiation field, relaxation of the muscle may occur due to the thermal effect. Furthermore, although during this study, the power density and radiation duration were relatively low, some temperature elevations occurred that are always expectable even to a little extent in low-level radiation [34]. Also, since trismus is a parameter that may be affected by pain [35], it can be expected that the better the pain management, the less the trismus. In many previous studies that benefited from low-level laser for this purpose, the researchers used the energy density of 40 J/cm^2^ [4, 31, 33]. It is because swelling does not decline postoperatively when the energy density is less than 4 J/cm^2^ [18, 36]. Hence, the energy density of 4 J/cm^2^ was applied for pain, swelling, and trismus alleviation. It is noteworthy that low-level laser radiation through the protein absorption by activating macrophages and regulating the intra-capillary pressure via reducing the permeability of vessels could lead to anti-inflammatory properties in this region [37]. It has also been emphasized that the time of applying low-level laser is very important, whether inflammation and the inflammatory processes have been initiated. As well, the stage at which the low-level laser is used is of importance [34]. It is well-established that inflammatory response is a typical postoperative process, and performing low-level laser radiation immediately after the surgery, at the beginning of the initiation of the inflammatory process, and when no swelling occurred is a typical and effective process in all studies to prevent the development of inflammation. Our study finding also showed that even with one session of radiation with no repetition, the swelling was less on the laser radiated side compared to the non-exposed side. Nevertheless, considering the method using which postoperative swelling was evaluated, the statistical data showed no significant difference between the two sides. In the present study, low-level laser radiation was intra orally performed, and possibly the most important reason for these different outcomes in a similar study can be considered to be the different applications of the laser as extra orally and intraoral in the two studies [22]. Nevertheless, many studies have mentioned better clinical effects of the extraoral application of laser in comparison with its intraoral usage and placebo [31]. In a study by Amarillas-Escobar et al., laser radiation was performed at six different points (both extraoral and intraoral points) in four sections [4]. Although the outcomes were reported as desirable, they were not statistically significant. However, the present study achieved the same desired results in one session of radiation that was performed immediately after the surgery. The results of this study are more valuable due to less waste of time and energy for both the patient and dentist. These wastes of time and energy are known to be among the undeniable disadvantages of multisession laser radiation studies over its advantages. Therefore, performing different studies is recommended to focus on low-level laser radiation in one session, so that by changing the different parameters of laser and medical conditions, better clinical outcomes that are statistically significant, are finally obtained. In the current study, the effects of demographic factors on the score of pain can be explained. The patients participating in this study had almost similar socioeconomic statuses and oral hygiene habits.

In some studies, no information on age and gender were given [6]. Many researchers have proposed that younger male patients have higher scores of pain [38, 39]. In the current study, there was no difference between the male and female patients regarding their pain score, but a slight difference was observed between the pain score reported by both the oldest and youngest participants. Furthermore, considering the varying degrees of hardness of operation, those patients with symmetric impacted teeth and with the same degree of hardness on both sides (Class A in Pell and Gregory classification regarding the impaction) were chosen to be included in this study. The lower proliferation of inflammatory mediators under non-inflammatory conditions and less proliferation of pain mediators would consequently reduce the pain sensation. Since in the split-mouth method, the decision on the selection of being a placebo or treatment case of the first surgery is based on randomness, pain severity control would be impossible after performing the second surgery, since the patient’s pain threshold would be changed after the first surgery [1]. In the present study, almost half of the patients included from the first surgery were placebo and the remained participants were experimental. Nevertheless, the clinical findings indicated that even in the patients who were placebo in the first surgery, the pain sensation was lower after performing the second surgery, suggesting the positive effect of the low-level laser radiation on pain mitigation which was statistically significant. Although some studies such as this study reported positive effects for laser energy, some of them reported no effect. These discrepant results might be due to the differences in the design of studies, different assessments in measuring postoperative swelling and trismus, usage of different lasers, various types of handpieces, and various radiation parameters [10, 18, 26, 32, 36].

Differences in the antibiotic and NSAID regimen between the two groups were included in the factors affecting the results [40]. To eliminate this effect, the same antibiotic regimen was given to all the included subjects, and as shown in Table 2, according to the patients’ recorded data, there was no significant difference between the two groups in the number of the consumed NSAIDs. The present study had some limitations. Firstly, several patients included during this study refused to continue the intervention and thus they were consequently excluded. Furthermore, the limited number of patients in this study, and usage of one laser wavelength were the other limitations. Therefore, it is suggested to perform more clinical trials in the future with larger sample sizes and with more diverse low-level laser wavelengths to examine the therapeutic effect of laser on pain, trismus, and mandibular or maxillary swelling following the surgical operation of the impacted third molar. Additionally, combining the current laser treatment with laser exposure on the masticatory muscles may more improve the trismus and opening of the mouth.

## Conclusions

In this clinical trial, the effect of intraoral low-level laser (500 mw) with a wavelength of 940 nm and an energy density of 10 J/cm^2^ was tested in one session after the surgical operation of the mandibular impacted third molar. The results reveal that LLLT has an alleviative effect on post-surgical pain. However, further studies are needed to improve the understanding of the effectiveness of LLLT on trismus and mouth opening by extraoral laser exposure on the masticatory muscles.

## Data Availability

The datasets used during the current study are available from the corresponding author on reasonable request.

## Abbreviations

COX-2: Cyclooxygenase-2
LLLT: Low-level laser therapy
NSAIDs: Nonsteroidal anti-inflammatory drugs
